# Barriers to seeking consultation for abnormal uterine bleeding: systematic review of qualitative research

**DOI:** 10.1186/s12905-020-00986-8

**Published:** 2020-06-12

**Authors:** Claire Henry, Alec Ekeroma, Sara Filoche

**Affiliations:** Department of Obstetrics, Gynaecology and Women’s Health, University of Otago, Wellington, New Zealand

**Keywords:** Abnormal uterine bleeding, Qualitative, Review, Women

## Abstract

**Background:**

Although Abnormal Uterine Bleeding (AUB) can have serious medical consequences and significantly impacts daily life, the overall trend is that most women do not seek care for these symptoms. The objective of this review was to synthesise factors impeding women’s access care for AUB.

**Methods:**

Systematic literature review of qualitative studies (interview and focus group) regarding the lived in experiences of women with abnormal menstrual symptoms, followed by a thematic analysis of these studies. We screened CINAHL, SCOPUS, ProQuest, OVID and Pubmed for qualitative studies. Studies were assessed using the Clinical Appraisal Skills Programme checklist and thematic synthesis was used to develop themes from the findings of the studies.

**Results:**

The review yielded 12 studies that satisfied the inclusion criteria. Three themes were developed that described barriers for women seeking care for AUB: health literacy (understanding of normal periods, role of cervical Pap smears and lack of access to appropriate information), taboo/normalisation (fear and embarrassment of symptoms, prioritising others) and health care provider (lack of accessible and trusted female GPs and poor experiences with GPs).

**Conclusions:**

For 20 years women have consistently reported poor experiences in accessing care for AUB. The findings from our review indicate that drivers to impeding access are multiple; therefore any approaches to improve access will need to be multi-level – from comprising local sociocultural considerations to improved GP training.

## Background

Abnormal uterine bleeding (AUB) is excessive, erratic and/or prolonged blood loss that interferes with a woman’s physical, social and mental quality of life. AUB may affect 10–30% of women of reproductive age [1, 2]. In the United States, it is estimated that 1.4 million cases of AUB are reported each year [3].

The International Federation of Gynaecology and Obstetrics (FIGO) clinically defines normal uterine bleeding as approximately 37-41mls of blood loss over the first 5–7 days of the menstrual cycle [4, 5]. FIGO defines heavy menstrual bleeding as 100-130mls of blood loss over varying number of days throughout the whole cycle but often within the first 10 in addition to anaemia. Recently, the FIGO system for classification and management of AUB has been reviewed with the aim to cease the use of poorly defined and confusing terms such as metorrhagia and dysfunctional uterine bleeding, and replaced them with terms that can be translated globally [6]. Most importantly, AUB can have a significant impact on women’s quality of social, emotional and mental health. A meta-analysis of US and European studies estimated that women with AUB have poor health related quality of life, below the 25th percentile of that of the general population [1]. It was also estimated that the annual direct and indirect costs of AUB to be upwards of $1 and $12 billion respectively [1]. AUB can be caused non-malignant conditions, such as infection, uterine fibroids, polyps, adenomyosis or endometriosis. However, it is also the most common symptom of Endometrial Cancer (EC) in post-menopausal women. In either situation, women with AUB should receive timely medical investigation [7].

Although AUB can have serious medical consequences and significantly impacts daily life, the overall trend is that most women do not seek care for these symptoms. In an international online survey study of 6179 women aged 18–55, 36% had either been diagnosed with heavy menstrual bleeding (HMB) or thought their menstruation was heavier than average [8]. 41% of these women believed there were no treatment options available for them. An internet survey conducted in Europe found that of 4506 *pre-*menopausal women, 27.2% experienced heavy menstrual bleeding (HMB), and 46% of these women had never sought medical consultation [9]. In addition, approximately 39.9% had experienced anaemic symptoms [9]. In New Zealand, approximately 37% of women who identify as Māori, and 23% of Non-Māori did not know that *post-*menopausal bleeding was abnormal, and around the same proportion did not know they needed to seek medical investigation [10]. In a Japanese study of 19,254 surveyed women, only 20% of those experiencing menstrual symptoms such as pain and heavy bleeding sought specialist consultation [11].

There is still a gap in understanding why majority of women are not accessing care for AUB. Qualitative studies based on interview data are important to gain an in depth understanding and inform strategies to improve support for women. Therefore the aim of this review was to synthesise the available qualitative evidence exploring the lived in experiences of women with AUB to identify barriers to seeking appropriate care.

## Methods

**Fig. 1:**
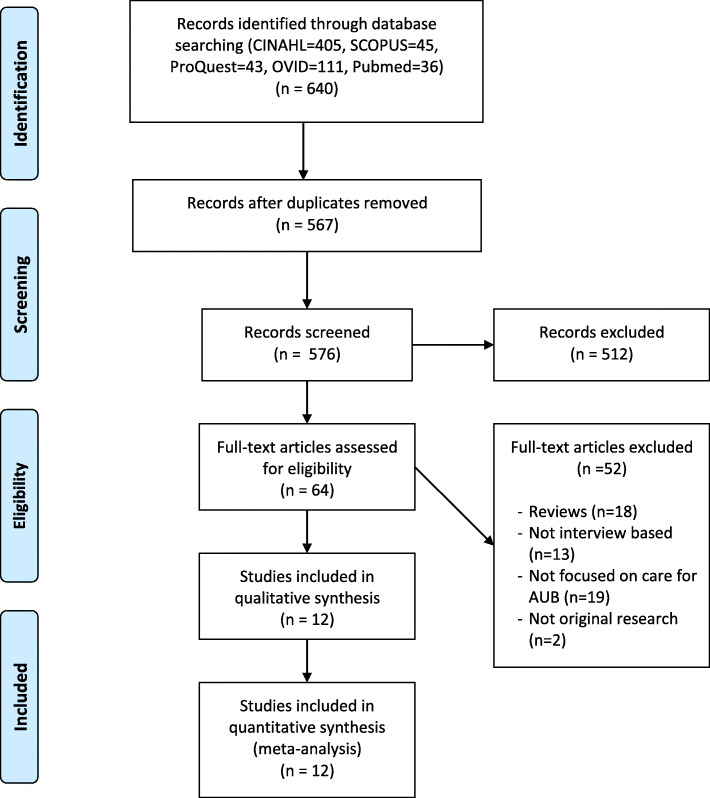
PRISMA flow chart of systematic search strategy and publication retrieval used to identify literature

A systematic search strategy and publication retrieval was used to identify literature (Fig. 1). CINAHL, SCOPUS, ProQuest, OVID (Medline) and PubMed databases were searched for the following key words (titles/abstracts):

- Abnormal uterine bleeding OR heavy menstrual bleeding OR menorrhagia OR gynaecological cancer OR endometrial cancer AND
- Experience OR qualitative OR perception OR perspective OR view OR need.

Date last searched was the 8th August 2019. Inclusion criteria: original qualitative study using interviews or focus groups with women aged over 18 years old. There were no limitations on study location or date of publication. Studies included were published in English. Twelve papers were retrieved which fit the criteria, dating from 1999 to 2018 and are listed in Table 1. Meta-ethnography methodology was used to report the synthesis of the qualitative studies (using translation and synthesis of key themes). The Clinical Appraisal Skills Program (CASP) Checklist was used to assess the quality of each study [24]. The CASP checklist for qualitative research outlines a set of 10 key points to address when critically analysing studies using methods such as surveys, interviews and focus groups.

**Table 1:** List of studies included in this review

Title	Location	Ref	Year
Menorrhagia: women’s perceptions of this condition and its treatment.	Manchester, England	[12]	1999
Menorrhagia in general practice – disease or illness.	London, United Kingdom	[13]	2000
What’s the delay? A qualitative study of women’s experiences of reaching a diagnosis of endometriosis.	London, United Kingdom	[14]	2006
Analysing qualitative data: health care experiences of women with gynaecological cancer.	Melbourne, Australia	[15]	2006
Menstrual symptoms: the importance of social factors in women’s experiences.	London, United Kingdom	[16]	2006
Women’s management of menstrual symptoms: Findings from a postal survey and qualitative interviews	Edinburgh, United Kingdom	[17]	2008
Gynaecologic cancer patients’ needs and experiences of supportive health services in New Zealand.	Auckland, New Zealand	[18]	2009
Questioning our questions: do frequently asked questions adequately cover the aspects of women’s lives most affected by abnormal uterine bleeding?	Rhode Island, USA	[19]	2010
Women’s interpretation of responses to potential gynaecological cancer symptoms: a qualitative interview study.	London, United Kingdom	[20]	2015
An altered perception of normal: understanding causes for treatment delay in women with symptomatic uterine fibroids.	Chicago, USA	[21]	2016
Exploring communication during the journey from noticing bodily changes to a diagnosis of endometria cancer.	Auckland, New Zealand	[22]	2017
A qualitative study of Pacific women’s knowledge and awareness of gynaecological cancers in Auckland, New Zealand.	Auckland, New Zealand	[23]	2018

## Results

Twelve papers were included for analysis. All passed the CASP checklist (summary provided in supplementary table 1). Qualitative studies ranged from open, unstructured and semi structured interviews with 16–60 participants. Six studies recruited women from community, and six recruited from attendance to clinical consultation (Table 2). All studies used a described method of qualitative analysis, such as thematic coding (Table 3).

**Table 2:** Characteristics of included studies: Participants

Ref	Diagnosis	Ages	***n***	Recruitment site
[12]	Menorrhagia	15–53	30	Community based
[13]	Menorrhagia	29–57	21	GP consultation
[14]	Endometriosis	16–47	32	Pelvic pain clinic
[15]	not given	not given	30	Gynaecology clinic
[16]	Menorrhagia	18–57	41	GP consultation and community
[17]	Menorrhagia	27–45	32	Community survey
[18]	Gynaecological Cancer	25–79	28	Gynaecology clinic
[19]	AUB	20–50	25	Gynaecology clinic
[20]	Gynaecological symptoms	30–69	26	Community survey
[21]	Fibroids	29–55	60	Community advertised
[22]	Endometrial Cancer	41–81	16	Gynaecology clinic
[23]	None	not given	20	Community advertised

**Table 3:** Characteristics of included studies: Methods

Ref	Data collection	Analysis	Findings
[12]	Semi structured interview	Thematic coding	Reality of problem, self-treatment, GP dissatisfaction.
[13]	Semi structured interview	Thematic coding and content analysis	Defining the problem, understanding of menstruation, causes of AUB, GP dissatisfaction.
[14]	Semi structured interview	Thematic coding	Understanding of normal periods, normalised by GP.
[15]	Un-structured interview	Grounded theory	Poor access to care, GP experience (normalised), understanding symptoms.
[16]	Semi structured interview	Constant comparative analysis	Pressure to conceal symptoms, social boundaries.
[17]	Un-structured interview	Constant comparative analysis	Self-treatment, concealment, resistance to see GP - dismissive and self-doubt.
[18]	Un-structured interview	Inductive thematic analysis	Need supportive care, resources available, participation in decision making.
[19]	Structured focus group	Thematic coding	Consistency in perception, social embarrassment. Built model of quality of life for clinical use.
[20]	Semi structured interview	Thematic coding	Normalising, self-management, competing demands, GP visits and gender.
[21]	Semi structured interview	Inductive thematic analysis	Perception of normal, limited knowledge, avoidance of GP.
[22]	Semi structured interview	Inductive thematic analysis	Assumptions, GP dissatisfaction, self-doubt, health literacy.
[23]	Structured interview	Thematic coding	Relationship and gender of GP, cost, stigma.

We identified three key themes across all papers in the context of barriers to women accessing care for abnormal or heavy menstrual bleeding.

### Theme 1: health literacy

Health literacy is the ability to interpret, maintain, understand and use health information to make informative health decisions and follow treatment instructions.

Half of the included studies identified general health literacy as a barrier to accessing care for AUB [12, 15, 19, 21–23]. One American study in 2010 noted the variability in 71 women in the perception of heavy and irregular bleeding [19]. Women described heavy bleeding by the number of pads used, the quality of the bleeding, and the length of bleeding [19]. The perception of blood loss was also described to be affected by the types of sanitary protection used [12]. Women found identification of ‘normal’ challenging, and if experienced for long enough, heavy or excessive bleeding became normalised and did not warrant the trouble of medical investigation. This was true in the Chicago study where the most commonly cited reason for delayed fibroid diagnosis was the perception that heavy bleeding was normal [21]. A United Kingdom study of 21 women found that explanations of ‘heavy’ bleeding were varied, including the appearance of blood and how it felt. In this study, women commented how it was difficult to describe experiences, particularly to someone who did not have similar feelings [13]. Only four women in this study had open discussions about periods with family or friends. In another study, even though women described discussing the ‘menstrual experience’ with others, heaviness of blood loss was not often brought up [17].

A study of Pasifika women’s knowledge and awareness of gynaecological cancer in New Zealand found that there was a need for culturally appropriate, easily accessible and correct information [23]. Many women in this study had never heard of the term gynaecology as there is no literal Tongan or Samoan translation of the word [23]. Women were aware of gynaecological cancers through personal experiences and relatives or friends that were diagnosed. This is re-iterated in another New Zealand study in women’s experiences with gynaecological support services where women expressed need for appropriate and timely information [18], and a United Kingdom study where half of the women who were diagnosed with uterine fibroids had never previously heard of the condition and expressed frustration they that lacked this knowledge [21].

Some women who developed their understanding of gynaecological conditions from family often had a confused interpretation of their symptoms. For example, in a United Kingdom study [20], many women believed gynaecological cancer symptoms such as abdominal size and irregular periods were due to factors such as diet, and managed themselves through avoiding certain foods:

*“ … I’d probably try and sort myself out first with eating and say right, that’s enough of dairy … ”*

In the same study, one woman disclosed that twins ran in her family, and that her heaviness and period pain was caused by release of two eggs during ovulation:

*“My nan reckons that a double egg comes from one side … that’s why I have been told that I get those pains”* [20]*.*

In a study of women previously diagnosed and treated for endometrial cancer [22], general uterine health and Pap smears were confused. Many women were upset that their routine cervical smear did not pick up EC:

*“It disappointed me … as long as you go for a smear test your fine”* [22]

This was echoed in a UK study, where eight of eleven women had considered AUB as a risk of cancer, but reassured themselves that a normal smear meant this was not the cause [13]. Whilst most women were aware of the need for routine Pap smears, confusion meant that women did not receive or perceive the correct information.

### Theme 2: taboo and normalisation

Gynaecological health has historically remained a taboo subject, yet this stigmatisation has meant that many women today are not able to openly talk about issues such as menstruation. This has resulted in many women normalising symptoms or ‘suffering in silence’.

Women may seek advice from friends to find reasons for normalisation and/or because they feel embarrassed or ashamed. For example, one woman noted:

“*I couldn’t talk to my mum because straight away she’d say to me ‘you have to go to the hospital”* [22]

Years of experience with menstrual cycles meant that for some women, change in vaginal discharge or spotting was no cause for alarm, especially when these symptoms varied from day to day. Over half of women with uterine fibroids minimised symptoms, by ‘suck (ing) it up and dealing with it’ [21]. Some women attributed their heavy periods to natural events, and assumed they were ‘unlucky’ in having bad ‘flow’ [13]:

“*There’s not a lot of point in reading or listening to anything, because it can’t be changed”* [13]

Women seemed to prioritise uterine/vaginal health lower than most other health issues, and ignored significant changes:

*“After I got out of bed the next morning it had eased off”* [22]*.*

“*It doesn’t happen the next day so you get on with your life”* [22]

Women were worried to seek medical consultation as they thought they would be ‘wasting their time’:

*“You tend to think you are wasting their time. You are not too sure whether it is happening to everybody …”* [12]

For those with excessive heavy bleeding, social embarrassment is a major determinant for discussing these issues, as many women provided examples of staining their clothes in public [19]. These experiences resulted in fear of social activities and avoidance of situations in which they felt ‘stranded’. For some, fear of leakage due to irregular timing and difficulties of management was a factor for women seeking seek consultation, particularly if these were increasing (which also shows how much women tolerated before they would seek help) [16, 20]. Yet for others, this held them back from seeking care:

“*I cancelled my doctor’s appointments for that reason, cause I bleed through everything. I’m afraid of sitting there and going through my clothes”* [19]

For women who identify as Pasifika, embarrassment of revealing ‘private parts’ during medical investigation was a big deterrent [23]. Many women felt uncomfortable with showing personal body parts, and find gynaecological examinations (pelvic exam) painful and scary:

*“I don’t even like seeing myself … that’s a huge barrier as to why I find it tricky accessing the doctor for smears or gynaecology troubles”* [23]

*“ … you know they may perform a test that hurts are very scary-if I can avoid it, I do”* [23]

### Theme 3: health care provider

Primary health care providers such as General Practitioners (GPs) are often the first medical point of contact for women with AUB. Therefore GPs have an important responsibility to take a thorough history and listen to all concerns to provide coordinated care with specialists when needed.

Most studies highlighted communication with health professionals as a key barrier to AUB investigation. Firstly, a regular or long term health care provider were viewed as preferential as these doctors know medical and family history [15, 22, 23]. Having an established and trusting relationship with a GP was found to be a positive facilitating factor for all women:

*“I have been with my GP for years and he knows what has happened to me … I just trust him”* [23]

Many women felt they could not speak to a male doctor about anything related to uterine health, and almost all women preferred to see a female doctor [20, 22, 23].

Surprisingly, eight of the 12 studies all identified normalisation and dismissal of women’s concerns by the health practitioner as an important barrier to accessing appropriate care [12–15, 17, 20–22] and was an issue that ran through studies from 1999 to 2017. In interviews with women 6–12 months post endometrial cancer surgery, a participant noted her symptoms were “brushed off” as a cause of menopause [22]. This attitude lead to women feeling reluctant to complain about symptoms, and didn’t want to ‘bother’ GP’s about their problems [17, 20]. In another, a woman’s period pain was dismissed, leading her to question the genuineness of her own experiences [14]. Women felt dissatisfied when doctors did not ask about how it was affecting their lives, their problem had not been given a name or had been explained vaguely, and felt that consultation had achieved little [13]. One woman found she had to ‘fight’ for treatment as her prolonged and heavy bleeding was impacting her relationship with her husband:

*“The woman [doctor] said these are things women have to put up with. I don’t think so. I won’t sacrifice my sex life”* [16]*.*

Two qualitative studies which were filtered in the selection criteria for this review were based on the perspective of health care providers. Supporting the challenges described, an American study found that of 417 GPs surveyed, 87% self-reported that they always ask a quality of life question (in relation to AUB) however only 17.5% ask a mood associated question. Only 18% of GPs thought that asking about quality of life was essential in evaluating women with AUB [25]. A United Kingdom study found that even GPs had difficulty in describing ‘normal’ periods. Female GPs reported that they were likely to ask details such as how many pads or tampons were used during a patient’s cycle. However, male GPs were less likely to go into this detail [26].

Incorrect diagnosis or inappropriate treatment was also described by women. In one study [16], several women had been prescribed norethisterone, and oral hormone treatment for their symptoms. At the time of the study (2006) GPs were advised not to prescribe this drug as it had been shown to not be effective at reducing blood loss. Worryingly, in two other studies of gynaecological cancer, many participants were given clinically irrelevant treatment following initial consultation for AUB:

*“I went back and forth … the doctors gave me tablets, nothing still wouldn’t stop”* [22]

*“You’re just being silly, you’re being paranoid”* [15]

*“My GP said I had an infection”* [15]

*“The doctor at the emergency said I had gastro”* [15]

The use of medical jargon by gynaecological specialists was noted in a number of studies, which left women feeling lost and fearful [15, 18, 22].

Logistics of attending appointments were often noted as barriers to seeking care, for example, long wait times, availability of doctors, and the demand of family, work and social commitments.

*“(GP) you ring now, you get an appointment in 3 weeks”* [22]

*“It would have to be easier to get an appointment with the GP. It really is that, that is such a bloody drama.”* [20]

*“I went to one … she had a baby … there was another doctor, then she left the clinic as well, then I’ve got doctor L. Now he’s only in every Wednesday”* [15]

*“It keeps moving down the list of priorities coz something else takes precedence.”* [20]

## Discussion

AUB is a medically difficult problem to diagnose and treat, which has lead to the recent development of investigation and management guidelines [6]. Whilst the amount and length of bleeding can be somewhat described and evaluated, women can have extremely different experiences based on their individual perceptions, symptoms and livelihoods. Factors such as normalisation, perception of health and relationships with others can impact the way in which the severity of AUB is viewed. AUB can be complex and non-specific, and underlying causes can often be left un-diagnosed – it was only in 2011 that a classification of underlying causes of AUB was published by FIGO [25]. This was reviewed again in 2018 [26]. This is likely due to the fact that AUB is not a healthcare priority, and has largely been under researched. The themes of GP dissatisfaction, self-treatment and lack of understanding around the severity of symptoms raised in the earliest paper from 1999 [12] were still present and of concern in the most recent papers [22, 23]. This suggests that women are continuing to have poor support for AUB and limited progress has been made to change these outcomes.

From the findings of this review, it is clear that there is limited information that is accessible to women, and what is currently available, lacks local socio-cultural considerations. Only one study asked women what they believe is needed to better support their health needs [23]. Specifically for New Zealand women who identified as Pasifika, the most important needs for them were: knowledge on what services are available for gynaecological issues, knowledge on the dangers of avoiding or delaying access to care and culturally appropriate information in their own language. A number of women from this study wanted services to be promoted and available in their community so they can be easily accessed, including during out of work hours [23]. The findings from this present review have highlighted that of the countries that were represented in the analysis, that access to appropriate support and services are limited, which has been reported elsewhere [27, 28]. Whilst there are successful awareness campaigns for women’s health in the context of cervical and breast cancer [29, 30], there are none for AUB, which effects at least four times as many women and can have severe life implications [3].

Primary health care management of AUB appears inadequate – for example misdiagnosis or dismissing of women’s symptoms. The reasons for this are likely many-fold, such as the definition of AUB being highly medicalised [4] and that the impact of AUB on women’s quality of life is not always viewed as an important metric, as noted by the experiences of women in the reviewed papers. A questionnaire-based study which explored health care provider views found that most physicians did not believe asking quality of life questions were essential in assessing AUB, and failed to properly assess the impact in a way that was meaningful to patients [31]. The study was limited to binary and multiple choice answers, and did not indicate why GPs felt this way. Ultimately, this creates a misalignment between what ‘medicine” sees as important, and what is important to women, and where women’s needs are not met. This is not specific for AUB, but for many other gynaecological problems, such as pelvic prolapse [32] and endometriosis [33]. There are moves to redress this misalignment, for example the National Institute for Health and Care Excellence (NICE, UK) published updated guidelines for the management of HMB in 2018 (NG88). These guideline updates were developed through processes of literature reviews, stakeholder engagement (including the public involvement program) and committee consultations. Here, the new guidelines list the impact of symptoms on quality of life as an integral part of history taking protocol and can help in the patient-preference decision making process for appropriate treatment options. Implementation of these guidelines by health professionals is recommended, with follow up surveys to assess the observance and impact on women’s health outcomes. It will be of great interest to see, and evaluate, whether such guidelines improve women’s access to appropriate treatment of AUB.

Taboo around the menstrual cycle has been long standing, influenced by a history of social and cultural stigma that entrenches modern life [34]. It is an overarching theme prevalent in every study in this review. In some countries, menstruation is rarely discussed in families and schools, and is often thought of as a disease, or impurity [35]. According to UNICEF, one third of girls in Asia do not know about menses, and half of girls in Pakistan do not have basic hygiene facilities or products [36]. Furthermore, men are taught less about menstrual cycles during school, and many feel it’s “not their problem” and regard it in a negative light, and these views are constructed through social, educational and relationship factors [37]. Some respondents in an interview based study indicated that during their school physical education classes, male students were removed from the lesson when menstruation was discussed [34]. The exclusion of men in discussions around menstruation can influence women’s health in their role as partners and family, and adds to the secrecy, taboo and separation of menstruation from everyday life [34]. Breaking the stigma of menstruation is an ongoing, global issue that will hopefully change with new generations and combating period poverty. It has been a topic that has gained international awareness over the last 10 years, with varying degrees of progress. Amnesty International prioritise campaigns to support women’s menstruation rights worldwide, and work to change the way menstruation is currently viewed [38]. In 2001, the WHO published a discussion document from the Madrid meeting ‘Mainstreaming gender equity in health: the need to move forward’, to improve health systems that are responsive to the needs of women, and delivers services and research that is inclusive of all women. In 2013, WHO released a statement on a new women’s health agenda, highlighting the need to address chronic conditions experienced by women, and the challenges needing to be overcome through social, system and policy efforts world-wide [39]. Outcomes from these campaigns have been slowly progressing. For example, some countries have implemented free pads and tampons provided at schools and colleges (such as the UK). However, in the US, menstrual product tax remains and exacerbates period poverty, decreasing the accessibility of basic health items for women [40].

### Strengths and limitations

Four studies were based on experiences with gynaecological cancers, one on experiences with endometrial cancer, four on opinions of women with AUB (including menorrhagia), one on endometriosis diagnosis, one on uterine fibroid diagnosis and one on general menstrual symptoms. Six of the studies were based in the United Kingdom, two from America and the remaining four from Australia or New Zealand. A limiting factor is that only studies published in English were included. In two studies, the ethnicity of women interviewed was not described [14, 19]. In 6 studies, over 60% of the participants identified as European [13, 16–18, 20, 22]. However in the remaining four studies, there was a representation of diversity amongst participants; one included women who identified as Pasifika [23], one included immigrant women of backgrounds such as Serbian and Croatian [15], one included mostly African American women [21] and another only included women of south Asian decent or Muslim faith [12]. Major themes were able to be elucidated across all studies, signifying common issues that women face with AUB.

There were a limited number of interview based studies on AUB and therefore papers spanning 20 years were included. This is a limitation of the study and identifies a gap in this area of research. However, similar themes were identified in papers published over 20 years ago, which indicates that barriers to care seeking behaviour have not changed in this time.

These studies capture a population of women that, whilst experiencing challenges with the accessing care, were able to seek and receive care. What these studies do not capture, is the population of women unable to access these pathways and are living day to day with debilitating pain and heavy bleeding, and some not directly associated with cancer. A community based approach would likely be more appropriate to engage women, with appropriate community consultation for example with community leaders, to gain a better understanding of the challenges faced by women who are not able to access care.

All studies passed CASP analysis (supplementary table 1), however all but one [19] did not address point 6 of the checklist – has the relationship between the researcher and participants been adequately considered? For example, did the researcher examined their own role and potential bias and influence on the formulation and response to research questions? This is a particularly important point for qualitative studies using interviews and should be taken into account in future studies. Whilst there is always risk of interviewer influence in these situations, consideration of these would need to be described in the ensuing dissemination of the study findings.

## Conclusion

There are a limited number of studies that have explored women’s experiences in accessing care for AUB, and those that have, report that women have poor experiences; the nature of which has not changed for over 20 years. Our findings indicate that improving access to care will require multi-level approaches that include consideration of local socio-cultural needs, along with improved training for primary healthcare providers such as GPs. AUB doesn’t just affect the lives of women, and more action needs to be taken to prioritise AUB as a healthcare priority in order to redress this area of unmet need.

## Supplementary information

**Additional file 1.**

## Data Availability

The datasets used and/or analysed during the current study are available from the corresponding author on reasonable request.

## Abbreviations

AUB: Abnormal Uterine Bleeding
FIGO: The International Federation of Gynaecology and Obstetrics
US: United States
UK: United Kingdom
EC: Endometrial Cancer
HMB: Heavy Menstrual Bleeding
CASP: Clinical Appraisal Skills Program
GPs: General Practitioners
PRISMA: Preferred Reporting Items for Systematic Reviews and Meta-Analyses
CINAHL: Cumulative Index of Nursing and Allied Health Literature
UNICEF: United Nations International Children's Emergency Fund
WHO: World Health Organisation
