# Supplementary material for: Barriers to seeking consultation for abnormal uterine bleeding: systematic review of qualitative research
Source: BMC Womens Health. 2020 Jun 12;20:123. doi: 10.1186/s12905-020-00986-8 (PMC7291434; doi:10.1186/s12905-020-00986-8)
Supplement: Supplementary file 1 — Additional file 1. [file 12905_2020_986_MOESM1_ESM.zip › Supplementary material_CASP checklist.docx]

| **REF** | **13** | **14** | **15** | **16** | **17** | **18** | **19** | **20** | **21** | **22** | **23** | **24** |
| --- | --- | --- | --- | --- | --- | --- | --- | --- | --- | --- | --- | --- |
| **Section A: Are the results valid?** |  |  |  |  |  |  |  |  |  |  |  |  |
| Was there a clear statement of the aims of the research? | Y | Y | Y | Y | Y | Y | Y | Y | Y | Y | Y | Y |
| Is a qualitative methodology appropriate? | Y | Y | Y | Y | Y | Y | Y | Y | Y | Y | Y | Y |
| Was the research design appropriate to address the aims of the research? | Y | Y | Y | Y | Y | Y | Y | Y | Y | Y | Y | Y |
| Was the recruitment strategy appropriate to the aims of the research? | Y | Y | Y | Y | Y | Y | Y | Y | Y | Y | Y | Y |
| Was the data collected in a way that addressed the research issue? | Y | Y | Y | Y | Y | Y | Y | Y | Y | Y | Y | Y |
| Has the relationship between researcher and participants been adequately considered? | N | N | N | N | N | N | N | Y | N | N | N | N |
|  |  |  |  |  |  |  |  |  |  |  |  |  |
| **Section B: What are the results?** |  |  |  |  |  |  |  |  |  |  |  |  |
| Have ethical issues been taken into consideration? | Y | Y | Y | N | Y | Y | Y | Y | Y | Y | Y | Y |
| Was the data analysis sufficiently rigorous? | Y | Y | Y | Y | Y | Y | Y | Y | Y | Y | Y | Y |
| Is there a clear statement of findings? | Y | Y | Y | Y | Y | Y | Y | Y | Y | Y | Y | Y |
|  |  |  |  |  |  |  |  |  |  |  |  |  |
| **Section C: Will the results help locally?** | Y | Y | Y | Y | Y | Y | Y | Y | Y | Y | Y | Y |

| *How valuable is the research?* | |
| --- | --- |
|  |  |
| ***13*** | One of the first to look at women's perspectives, stemming from previous GP centric study. |
| ***14*** | Argues for a revaluation of the model of menorrhagia used in clinical practice. |
| ***15*** | Also included quantitative data - time to referral and diagnosis. |
| ***16*** | Raises issues of health literacy and gynaecological symptom knowledge. |
| ***17*** | Uses 2 sets of interviews - first participants recruited from GP interviewed to develop themes, then recruited from community. |
| ***18*** | Constraining women's beliefs with regard to menstrual symptoms may deny them the information, advice or treatments they need. |
| ***19*** | More support needed, community services, participation in decisions. |
| ***20*** | Consistency in perception, social embarrassment. Developed model of quality of life for clinical use |
| ***21*** | Highlights self-management techniques and women adopting a lay system of care. |
| ***22*** | Designed women’s health belief model from results. |
| ***23*** | Highlights erratic trajectory towards endometrial cancer diagnosis. Issues around perceived protection from cervical smears. |
| ***24*** | Important for Pacific women’s health research and highlights local barriers. |
